# High-Frequency Weak Magnetic-Field Detection and Dose Calibration for Micro-Magnetic Stimulation: A Review

**DOI:** 10.3390/s26154807

**Published:** 2026-07-28

**Authors:** Lei Tian, Hang Tu, Jiahao Fei, Shuai Cao, Yongxiang Gao, Yameng Zhou, Jianpeng Song

**Affiliations:** 1 School of Control Science and Engineering, Tiangong University, Tianjin 300387, China; 2531091232@tiangong.edu.cn (H.T.); 2431091183@tiangong.edu.cn (J.F.); 2 School of Life Sciences, Tiangong University, Tianjin 300387, China; 2531131490@tiangong.edu.cn (S.C.); 2531131493@tiangong.edu.cn (Y.G.); 2431131406@tiangong.edu.cn (Y.Z.); 2431131387@tiangong.edu.cn (J.S.)

**Keywords:** micro-coil, high-frequency weak magnetic-field detection, dose calibration, resonance detection, μMS

## Abstract

High-frequency weak magnetic fields generated by submillimeter micro-coils are key physical parameters associated with stimulation dose in micro-magnetic stimulation (μMS). However, their low amplitude, highly localized near-field distribution, frequency-dependent probe response, and sensitivity to coupling variations make accurate measurement and dose calibration challenging. This review summarizes recent advances in high-frequency weak magnetic-field detection techniques for micro-coil systems. It discusses key measurement challenges, including probe parasitics, impedance mismatch, phase distortion, spatial averaging, and dynamic detuning. Existing methods are classified into direct magnetic-field measurement, resonance-enhanced detection and calibration, spatial-field mapping reconstruction, and adaptive compensation. These methods are compared across five dimensions: frequency range, sensitivity, spatial resolution, system complexity, and online integration capability. The review further highlights the need to establish reliable and traceable relationships among detector outputs, local magnetic fields, and stimulation dose. Future directions include broadband adaptive measurement, online dose calibration, multichannel magnetic-field imaging, multichannel magnetic-field component decoupling, and physics-constrained data-driven field estimation. Overall, this review provides a structured overview of micro-coil magnetic-field measurement, stimulation dose standardization, and the quantitative frameworks for closed-loop neuromodulation microsystems.

## 1. Introduction

High-frequency weak magnetic-field detection is a fundamental measurement technique for micro-coil-based micro-magnetic stimulation (μMS), implantable biomedical electronics, and related microscale electromagnetic systems [1,2,3]. As these systems become increasingly miniaturized and integrated and operate at higher frequencies, magnetic-field measurement must address weak field amplitudes, localized magnetic-field distributions, complex coupling conditions, and frequency-dependent probe responses [4,5,6,7,8]. In the kHz–MHz range, probe parasitics, phase distortion, impedance mismatch, and external electromagnetic interference can further degrade measurement accuracy. Therefore, accurate detection of local mT-level or lower magnetic fields is essential for performance evaluation and dose calibration in high-frequency micro-coil-based μMS systems.

High-frequency weak magnetic-field detection has been addressed using various measurement approaches, including inductive coils, near-field magnetic probes, solid-state magnetic sensors, resonance-based detection structures, and precision magnetic-field metrology. Among them, inductive methods offer a passive, structurally simple approach to measuring alternating magnetic fields. However, in millimeter- or submillimeter-scale micro-coil measurements, their accuracy is strongly affected by probe parasitics, self-resonance, probe-area-induced spatial averaging, and phase distortion [4,5,7,9,10,11]. Solid-state magnetic sensors, such as Hall, anisotropic magnetoresistive (AMR), and tunneling magnetoresistive (TMR) sensors, offer high static sensitivity and miniaturization potential. However, their limited bandwidth and the robustness requirements of associated readout circuits constrain their application in MHz-range alternating magnetic-field measurements [12,13,14]. Precision metrology techniques, including superconducting quantum interference devices (SQUIDs), atomic magnetometers, nuclear magnetic resonance (NMR), and optical magnetic sensing, provide high-accuracy reference measurements. However, their complex operating conditions and costly instrumentation make them better suited to standard calibration or offline validation [15,16,17,18,19,20]. Beyond device-level optimization of micro-coils, probes, and measurement electronics, material-level engineering of magnetic responses represents another potential strategy for improving weak magnetic-field sensing capability. Recent studies on perovskite manganites have demonstrated enhanced room-temperature magnetoresistive and magnetocaloric responses associated with Griffiths-phase-related ferromagnetic clusters above the Curie temperature [21]. Although these magnetic functional materials are not directly applied to micro-coil field detection, they provide valuable insights into magnetic-response modulation. They may inspire future high-sensitivity sensing elements for measuring weak magnetic fields.

Although progress has been made in inductive measurement, mutual-inductance modeling, resonance-enhanced detection, spatial field scanning, and adaptive tuning, these approaches differ in their measurement principles, advantages, and application scenarios. Inductive measurement provides a simple and direct approach for converting time-varying magnetic flux into a measurable voltage, making it suitable for basic magnetic-field characterization [22,23]. Mutual-inductance modeling establishes an analytical relationship among coil geometry, relative position, induced voltage, and coupling strength, and is therefore useful for calibration and coupling analysis [24,25]. Resonance-enhanced detection improves sensitivity to weak narrowband magnetic fields [26,27], whereas spatial field scanning enables two- or three-dimensional mapping of localized near-field distributions [28,29,30]. Adaptive tuning further improves measurement stability by compensating for frequency drift, impedance mismatch, and coupling variations [31,32].

However, these studies remain scattered across different measurement principles and application scenarios. A systematic review focused on high-frequency weak magnetic-field detection and dose calibration in micro-coils is still lacking. This review, therefore, summarizes the key challenges associated with MHz-range frequency response, μT–mT weak-field resolution, probe-area-induced spatial averaging, multi-frequency and multichannel coupling, and online dose consistency. Existing methods are classified and compared from three perspectives: direct signal acquisition, resonance enhancement and precision calibration, and spatial field reconstruction with dynamic compensation. Based on this classification, their applications in magnetic-coupling-resonance wireless power transfer (MCR-WPT), implantable micro-coil systems, and multichannel neuromodulation are further discussed. Finally, this review aims to provide a methodological reference for selecting microscale magnetic-field measurement strategies, standardizing magnetic stimulation dose, and designing next-generation closed-loop neuromodulation microsystems.

## 2. Requirements and Challenges in High-Frequency Weak Magnetic-Field Detection for Micro-Coils

Strong spatial localization, high field gradients, and rapid attenuation over submillimeter distances typically characterize the magnetic fields generated by micro-coils. Compared with macroscale uniform magnetic fields, micro-coil magnetic-field measurements are more strongly affected by probe geometry, detection area, measurement distance, and spatial orientation. The frequency-dependent probe response further introduces measurement uncertainty. Therefore, detecting high-frequency, weak magnetic fields generated by micro-coils requires specialized methods rather than conventional approaches designed for low-frequency or large-scale magnetic-field measurements.

The quantitative design of microscale electromagnetic systems and the calibration of stimulation dose pose several key challenges for researchers. These challenges include accurate measurement of amplitude and phase in MHz-range alternating magnetic fields, high-signal-to-noise detection of μT–mT-level weak magnetic fields, and spatial characterization of submillimeter near-field distributions. Additional challenges involve decoupling multi-frequency and multi-channel composite fields, as well as performing online calibration of magnetic-field dose under complex operating conditions.

Considering the diversity of measurement principles and performance metrics reported in previous studies, several key measurement-related terms are defined to establish consistent evaluation criteria in this review. In micro-coil applications, a weak magnetic field is typically defined as a localized field ranging from μT to mT, where reliable detection is affected by sensor noise, probe characteristics, electromagnetic interference, and calibration uncertainty [4,5,6,7,8]. Weak-field resolution denotes the minimum distinguishable variation in magnetic-field strength under specified measurement conditions. Detection sensitivity represents the change in measurement output per unit change in magnetic-field strength, whereas field response describes the measurable output characteristics induced by an applied magnetic field, including amplitude, phase, gain variation, or resonance shift. Sensitivity, resolution, minimum detectable field, and noise spectral density are considered distinct performance indicators and are interpreted according to the definitions used in the corresponding studies.

### 2.1. Bottlenecks in MHz-Range High-Frequency Magnetic-Field Measurement

When a micro-coil system operates in the kHz–MHz range, measurement accuracy is strongly affected by the frequency-dependent amplitude and phase responses of the sensing probe and signal-conditioning circuits. Impedance mismatch, parasitic parameters, and probe self-resonance can further introduce additional measurement errors. According to Faraday’s law of electromagnetic induction, the electromotive force induced in an *N*-turn detection coil is determined by the time derivative of the magnetic flux linkage [23]. Assuming that approximately the same magnetic flux links each turn of the detection coil, the induced voltage can be expressed as

(1) $$V_{\mathrm{emf}}\left(t\right)=-N\frac{d\Phi (t)}{dt}=-N\frac{d}{dt}\int_{S_{\mathrm{eff}}}B\left(r,t\right)\cdot da$$

where $V_{\mathrm{emf}}\left(t\right)$ represents the induced electromotive force; Φ(t) represents the magnetic flux through one turn of the detection coil; $S_{\mathrm{eff}}$ denotes the effective sensing surface; B(r,t) represents the spatially varying magnetic flux density; and da denotes the differential area vector normal to the coil surface. For a stationary probe with a constant effective area and orientation, the induced voltage can be approximated as

(2) $$V_{\mathrm{emf}}\left(t\right)\approx -NA_{\mathrm{eff}}\frac{dB_{n}\left(t\right)}{dt}$$

where $A_{\mathrm{eff}}$ is the effective sensing area and $B_{n}\left(t\right)$ denotes the component of the magnetic flux density normal to the coil surface. However, this uniform-field approximation may become inaccurate for highly localized micro-coil near fields, where the magnetic flux density varies significantly across the finite probe area. Therefore, the full flux-integral formulation should be retained to account for the spatial averaging caused by the finite probe size.

**Figure 1:**
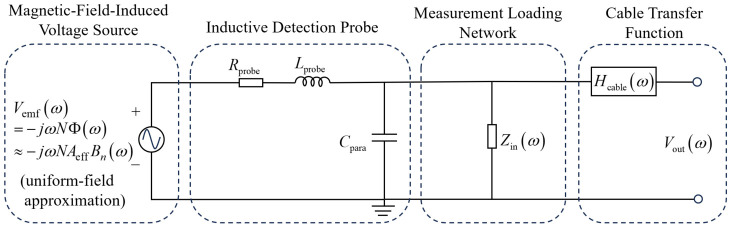
Lumped-equivalent circuit of a high-frequency inductive magnetic-field measurement chain, including the induced voltage source, probe parasitic elements, measurement loading, and cable transfer function.

Beyond spatial-averaging effects, high-frequency operation introduces additional circuit-level limitations. As illustrated in Figure 1, under MHz-range excitation conditions, the detection probe can no longer be treated as an ideal voltage source, and the frequency response of the probe and readout chain must be considered.

Based on the lumped equivalent circuit shown in Figure 1, the measured output voltage is determined not only by the induced electromotive force $V_{\mathrm{emf}}\left(\omega \right)$ but also by the probe resistance $R_{\mathrm{probe}}$, self-inductance $L_{\mathrm{probe}}$, inter-winding parasitic capacitance $C_{\mathrm{para}}$, equivalent input impedance $Z_{\mathrm{in}}\left(\omega \right)$, and cable-transfer function $H_{\mathrm{cable}}\left(\omega \right)$. These parameters jointly form a frequency-dependent RLC network that may exhibit self-resonance [33,34].

The measured output voltage can therefore be expressed as

(3) $$V_{\mathrm{out}}\left(\omega \right)=V_{\mathrm{emf}}\left(\omega \right)\frac{Z_{\mathrm{eq}}\left(\omega \right)}{R_{\mathrm{probe}}+j\omega L_{\mathrm{probe}}+Z_{\mathrm{eq}}\left(\omega \right)}H_{\mathrm{cable}}\left(\omega \right)$$

where $Z_{\mathrm{eq}}\left(\omega \right)$ represents the parallel combination of $Z_{\mathrm{in}}\left(\omega \right)$ and the inter-winding parasitic capacitance. This relationship indicates that the measured voltage is determined not only by the external magnetic field but also by the intrinsic electrical characteristics of the probe and the signal-acquisition chain.

When the operating frequency approaches the probe self-resonant frequency, parasitic capacitance, impedance loading, and cable-transfer effects introduce increasingly significant amplitude and phase distortions. Therefore, high-frequency magnetic-field measurements require frequency-response characterization, calibration, and compensation rather than direct voltage-to-field conversion. Chen et al. [4,5] achieved electromagnetic induction-based measurements of μT-level magnetic fields in the 0.5–1.5 MHz range, with a relative error of approximately 2.5%, demonstrating the feasibility of conventional inductive methods under specific high-frequency conditions. However, these validations were primarily conducted using relatively large coils or in controlled magnetic-field environments. Therefore, their conclusions may not be directly applicable to near-field measurements of millimeter- or submillimeter-scale micro-coils.

For millimeter-scale micro-coils, frequency-dependent reactive impedance at high frequencies can degrade the correspondence between the detected voltage and the actual magnetic-field strength. The main limitations include insufficient induced-voltage sensitivity, probe-area-induced spatial averaging, parasitic-parameter-related amplitude distortion, and phase delay. Tang et al. [7] applied the conventional Faraday-induction-based measurement method to a 13.56 MHz wireless power-transfer system, with a measurement error of 11.45%. This result indicates that non-resonant inductive measurements may suffer from significant systematic deviations under high-frequency operating conditions. These deviations can be reduced through appropriate frequency-response calibration, phase correction, and reactive-impedance compensation.

To improve the sensitivity of weak magnetic-field detection, an external tuning capacitor $C_{\mathrm{tune}}$ can be connected in parallel with the detection probe to establish resonance near the target operating frequency. By combining the tuning capacitor with the intrinsic inter-winding parasitic capacitance $C_{\mathrm{para}}$, the equivalent capacitance is given by $C_{\mathrm{eq}}=C_{\mathrm{para}}+C_{\mathrm{tune}}$. Assuming a high input impedance and relatively small circuit losses, the probe exhibits parallel resonance when the inductive and capacitive susceptances cancel:

(4) $$1-\omega_{0}^{2}L_{\mathrm{probe}}C_{\mathrm{eq}}=0,\;\omega_{0}=\frac{1}{\sqrt{L_{\mathrm{probe}}C_{\mathrm{eq}}}}$$

Equation (4) describes the parallel resonance behavior of the inductive detection probe rather than a series-resonant configuration. Under this condition, the LC resonant network can enhance the induced output voltage, with the enhancement determined by the loaded quality factor and circuit loading conditions. The loaded quality factor $Q_{L}$ can be estimated as

(5) $$Q_{L}=\frac{f_{0}}{\Delta f_{3\mathrm{dB}}}$$

where $f_{0}$ is the resonant frequency and $\Delta f_{3\mathrm{dB}}$ is the −3 dB bandwidth. A higher $Q_{L}$ indicates a sharper resonance response and generally provides stronger voltage enhancement and improved weak-field sensitivity. However, it also reduces the measurement bandwidth and increases sensitivity to frequency drift, loading variation, and coupling-state perturbations.

Based on this resonance-enhancement principle, several studies have explored resonant and magnetically coupled structures to improve weak magnetic-field detection in micro-coil systems. Tian et al. developed a mutual-inductance model for millimeter-scale planar coils and achieved weak magnetic-field measurements within the range of 0–3.06 mT [1,6]. Liu et al. further proposed a three-stage magnetic-coupling structure, enabling mT-level magnetic-field measurements in the 920 kHz–2 MHz range [2]. These studies indicate that resonant enhancement and magnetic coupling are useful for extending weak magnetic-field detection to micro-coil systems. However, existing approaches still largely rely on offline parameter calculation and manual frequency matching, resulting in limited online adaptability. Therefore, MHz-range micro-coil magnetic-field detection should be further integrated with hardware-level adaptive methods. These methods include reactance compensation, frequency tracking, and online impedance correction, which can improve measurement stability under complex operating conditions.

### 2.2. Dose Calibration and Dynamic Tuning Under Complex Conditions

In this review, “magnetic dose” is defined as an operational concept used to describe the magnetic stimulation delivered by a micro-coil system. It is not limited to the instantaneous magnetic-field amplitude but also includes the spatial distribution of the field, operating frequency, waveform characteristics, pulse width, duty cycle, and exposure duration. Additional delivery conditions, such as coil position and coupling conditions, may further influence the actual delivered dose. This definition follows the general principle in electromagnetic neuromodulation that stimulation dose is determined by multiple parameters rather than a single intensity value. In these applications, stimulation dose is typically determined by multiple parameters, including device configuration, stimulation intensity, pulse waveform, repetition frequency, train duration, total number of pulses, and total exposure time [35,36].

This operational definition enables the classification of magnetic dose into three related levels. The physical dose refers to the measurable electromagnetic stimulation parameters generated by the coil system, including local magnetic-field strength, frequency, waveform, and exposure duration. The biological effective dose refers to the effective stimulation component that produces measurable physiological responses, such as changes in neuronal firing, local field potentials, field excitatory postsynaptic potentials, or synaptic plasticity responses. The cumulative dose is the total delivered exposure over time, determined by pulse number, duty cycle, stimulation-train duration, repeated sessions, and total exposure time. Therefore, magnetic-dose calibration for micro-magnetic stimulation should quantify not only the local magnetic-field amplitude but also the temporal structure and cumulative exposure of the stimulation.

The magnetic-field strength generated by micro-coils is typically in the μT–mT range. In μMS and neuromodulation applications, magnetic-field strength is not merely an output metric of the device but is closely associated with neuromodulation thresholds, stimulation safety margins, and experimental reproducibility [37,38,39,40]. Zheng et al. showed that neuronal excitability and synaptic plasticity responses exhibit dose-dependent changes in weak mT-level magnetic stimulation [41]. Therefore, accurate calibration of high-frequency weak magnetic fields is essential for establishing the relationship between magnetic-stimulation dose and neural response [42].

Existing magnetic-field detection methods still face challenges in simultaneously achieving an MHz-range frequency response and sub-mT weak-field resolution. Among existing magnetic-field measurement approaches, conventional transcranial magnetic stimulation (TMS) magnetic-field measurement methods can provide spatially resolved measurements over large three-dimensional regions. For example, Liu et al. developed a field-programmable gate array (FPGA)-based multichannel real-time magnetic-field detection system with an operating frequency range of 0–20 Hz [43]. However, this frequency range is far below the MHz range required for high-frequency micro-coil systems. Song et al. achieved a high sensitivity of 52.17 nT at 500 kHz through strong coupling and magnetic induction, indicating that structural optimization can improve weak-field resolution [8]. However, for neuromodulation micro-coils, high sensitivity alone is insufficient for reliable magnetic-field measurement. The detection system must also address microscale positioning accuracy, high-frequency response, and measurement uncertainty caused by dynamic coupling variations [44,45,46].

Because micro-coil magnetic fields are highly localized, the measured signal can be strongly affected by probe size. A probe with a size comparable to or larger than the target stimulation region primarily detects area-averaged magnetic flux rather than the local magnetic field at the target position. This spatial averaging can underestimate the peak field and distort dose-distribution reconstruction. Tian et al. developed a wireless passive micro-magnetic stimulation (WP-μMS) system based on a planar spiral micro-coil that operates at 920 kHz and generates a magnetic stimulation intensity of 1.15 mT [47]. Because the stimulation field varies significantly at the microscale, reliable field detection requires careful control of probe size, measurement distance, and three-dimensional positioning accuracy. In contrast, the TMS probe designed by Meng et al. [48] has a diameter of 4 mm and a height of 5 mm and was primarily developed for three-dimensional vector-field measurements of TMS coils. Therefore, directly applying this probe to micro-coil magnetic-field measurements remains challenging.

In multi-target, multi-channel, and multi-frequency neuromodulation microsystems, the measured magnetic field becomes a composite field with overlapping spatial and spectral components. Under these conditions, inter-channel coupling, variations in reflected impedance, and load changes may further induce resonant-frequency shifts. Adaptive tuning and active reactance compensation in wireless power transfer (WPT) provide potential solutions for mitigating dynamic detuning in coupled micro-coil systems. Representative adaptive-tuning and reactance-compensation methods, such as the automatic tuning assist circuit (ATAC) and synchronous series compensation, have been used to improve system stability with frequency drift, coupling variation, and changes in reflected reactance [49,50,51,52]. These studies suggest that magnetic-field measurements in multi-frequency, multi-channel micro-coil systems should move beyond fixed-parameter offline calibration. Future multichannel decoupling strategies should integrate spectral analysis, phase compensation, and dynamic impedance tuning.

In summary, high-frequency weak magnetic-field detection in micro-coils is affected by multiple factors, including amplitude and phase distortions, spatial averaging caused by the finite probe area, and multichannel coupling. Dynamic detuning and the limited adaptability of offline calibration further increase measurement uncertainty under complex operating conditions. Future detection systems should evolve from offline, static measurements toward dynamic frameworks that integrate real-time detection, dose estimation, online calibration, and closed-loop correction. By integrating physics-based models, data-driven prediction, and closed-loop calibration, future frameworks may further improve the accuracy and consistency of microscale magnetic-field dose assessment under complex operating conditions.

## 3. Methods for High-Frequency Weak Magnetic-Field Detection

**Figure 2:**
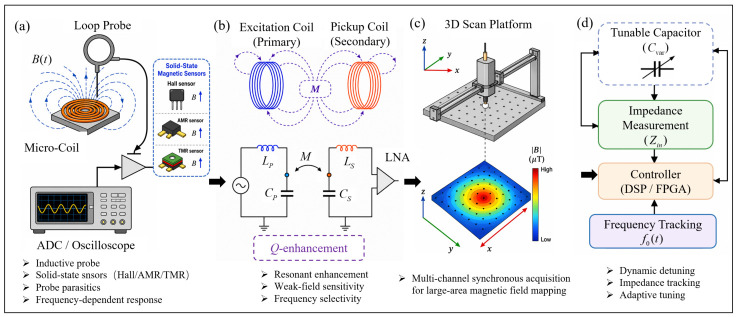
Technical roadmap for high-frequency weak magnetic-field detection in micro-coil systems. The figure summarizes four representative approaches: (**a**) direct magnetic-field measurement using inductive probes, solid-state magnetic sensors, and electrical readout; (**b**) resonance-enhanced detection based on magnetically coupled resonators; (**c**) spatial field mapping via 3D scanning and multichannel acquisition; and (**d**) active tuning and compensation using impedance feedback and frequency tracking. These methods provide a unified framework for weak-field measurement, spatial characterization, and system calibration in micro-coil systems. Abbreviations: ADC, analog-to-digital converter; LNA, low-noise amplifier.

In micro-coil systems, high-frequency weak-field detection should be regarded as a coupled measurement problem involving probe geometry, electrical response, microscale magnetic-field characteristics, and operating conditions, rather than a simple choice of sensor type. Therefore, classifications based solely on sensor type, such as inductive coils, Hall sensors, magnetoresistive sensors, and optical magnetometers, are insufficient for micro-coil magnetic-field measurement. They cannot fully describe the signal-acquisition mechanisms, coupling-enhancement strategies, and dose-calibration requirements involved in this problem. Considering the practical measurement conditions of high-frequency weak-field detection and the dose-evaluation requirements of neuromodulation applications, this review categorizes existing methods into four representative technical routes based on their measurement principles and system functions: direct magnetic-field measurement [4,5,9], spatial field mapping [43,48,53], resonance-enhanced detection [6,54,55], and active tuning and compensation [49,50,51,52]. These routes are summarized in Figure 2 and serve as the organizational framework for the following subsections.

### 3.1. Direct Magnetic-Field Measurement Methods

As illustrated in Figure 2a, direct magnetic-field measurement converts the magnetic field generated by a micro-coil into a measurable electrical signal using inductive probes or solid-state magnetic sensors. This approach provides a straightforward means of characterizing magnetic-field amplitude, frequency, and local spatial variation. It is commonly used for pointwise field measurement, basic system calibration, and probe–coil coupling analysis. However, its accuracy depends strongly on probe dimensions, orientation, frequency response, parasitic parameters, and the spatial distribution of the target field.

Direct magnetic-field measurement methods are primarily based on Faraday’s law of electromagnetic induction or on solid-state magnetic effects. Among these, inductive detection is widely used for high-frequency weak-field measurements because it uses a structurally simple passive probe and directly responds to alternating magnetic fields. Chen et al. [4,5] demonstrated MHz-range magnetic-field measurements using Faraday induction coils. However, for highly localized micro-coil fields, inductive detection is limited by spatial averaging over the finite probe area and by amplitude and phase distortions caused by high-frequency parasitic effects. Although Huang et al. [9] achieved measurement errors of 5% using a 400-turn Helmholtz coil with a radius of 0.1 m, this macroscale configuration does not account for spatial averaging in microscale field measurements.

For millimeter-scale magnetic-field measurements, mutual-inductance modeling is essential for compensating for variations in probe position, orientation, and coil misalignment. To address misalignment, Joy et al. [10] proposed an analytical method to calculate the mutual inductance between coplanar rectangular coils. Building on this work, Raju et al. [56] developed a mutual-inductance model that accounts for both axial and lateral coil displacements. Further building on this work, Wu et al. [11] subsequently derived an analytical solution for rectangular coils with varying cross-sectional aspect ratios. Their solution limited the deviation in mutual-inductance calculations to less than 3%. These models provide a theoretical basis for estimating micro-coil magnetic fields and compensating for probe-position deviations and orientation errors.

Solid-state magnetic sensors, including Hall, AMR, and TMR sensors, are characterized by compact device structures and high static sensitivity. They have been widely used for position tracking, static-field calibration, and low-frequency weak-field detection. Zhang et al. [13] used a 1.0 mm×1.0 mm AMR sensor for magnetic-gradient-based positioning over a 50–140 cm range, achieving a relative positioning deviation below 10%. Li et al. [14] developed a high-density Hall sensor array, reducing static-field measurement deviation to within 0.1%. For weak-field resolution, Song et al. [8] proposed a magnetic-film/planar-coil multilayer heterogeneous sensor, achieving a magnetic-field resolution of 52.17 nT at 500 kHz. These studies suggest that material design and coil-structure optimization can improve weak-field resolution.

Giant magnetoimpedance (GMI) sensors provide a promising approach for extremely weak magnetic-field detection. In soft magnetic wires, ribbons, or thin films excited by a high-frequency current, an external magnetic field changes the transverse permeability and consequently the electrical impedance. GMI sensors have been investigated for biomagnetic measurements, including muscular magnetic activity, magnetocardiography, and magnetoencephalography [57,58]. For example, Han et al. developed a dual-loop self-adaptive GMI gradiometer that achieved background noise levels of $12\;\mathrm{pT}/\sqrt{\mathrm{Hz}}$ at 10Hz and $7\;\mathrm{pT}/\sqrt{\mathrm{Hz}}$ at 200Hz in an unshielded environment [59]. GMI-based sensors have also been used to detect weak stray fields generated by magnetic particles or nanoparticles in biological samples and tissue-scale environments [60]. However, their performance depends on the bias condition, excitation parameters, environmental magnetic noise, and readout-electronics stability [58,61].

Despite their different sensing mechanisms and sensitivity ranges, the application of conventional Hall, AMR, and TMR sensors, as well as high-sensitivity GMI sensors, to MHz-range micro-coil magnetic-field measurement remains challenging. First, the high-frequency response of solid-state magnetic sensors is limited by material dynamics and the bandwidth of the readout circuit. For example, Zaini et al. [12] developed a 6mm×20mm probe capable of measuring magnetic fields in the mT range. However, its operating frequency range is limited to 110–210 Hz, so this approach is not directly applicable to micro-coil magnetic-field measurements in the MHz range. Second, Hall, AMR, and TMR sensors typically require biasing and signal-conditioning circuits, including amplification and filtering stages. In strong high-frequency electromagnetic fields, these circuits may be affected by electromagnetic coupling noise, front-end saturation, and parasitic signal paths [62]. Although Meng et al. reported a 17mm×17mm×3mm miniature Hall sensor with a resolution of 0.1477 μT, its stability in MHz-range magnetic fields has yet to be validated [48]. Similarly, although GMI sensors offer substantially higher weak-field sensitivity, their dependence on high-frequency excitation, bias conditions, and low-noise readout electronics may complicate their direct use near strongly coupled MHz micro-coil systems. Therefore, Hall, AMR, TMR, GMI, and related solid-state magnetic sensing methods are generally more suitable for low-frequency auxiliary calibration, static-field mapping, or spatial positioning, biomagnetic-field detection, or magnetic-particle sensing. In contrast, their use for MHz-range weak-field detection in multifrequency and multichannel neuromodulation micro-coil systems remains to be fully validated.

### 3.2. High-Precision Magnetic-Field Calibration and Resonance-Enhanced Detection

As illustrated in Figure 2b, resonance-enhanced detection employs resonant structures to amplify weak magnetic-field responses near the target frequency. Resonance-enhanced detection improves weak-field sensitivity by increasing the probe response through frequency-selective resonance. High-precision magnetic-field metrology, including SQUIDs, atomic magnetometers, NMR, and optomechanical magnetic sensing, provides reference standards for calibration and validation, whereas resonance-enhanced approaches offer a more practical solution for compact micro-coil measurement systems.

Precision magnetic-field metrology, including SQUIDs, atomic magnetometers, NMR, and optomechanical magnetic sensing, can serve as a high-precision reference tool for weak-field calibration. In contrast, resonance-enhanced detection improves weak-field sensitivity by increasing the probe response near the target frequency through frequency-selective resonance. Dong et al. [15] developed an NMR imaging system based on a high-temperature SQUID magnetometer and achieved precise measurement of a 212.2 μT magnetic field at 9.038 kHz. However, such systems require cryogenic operation and are associated with a large system size and high maintenance costs. Other precision metrology approaches have also been reported, including rubidium-vapor-based measurement of strong pulsed magnetic fields above 1 T [16], NMR probe-array calibration over the 200 mT–30 T range [17], flux-modulation- and lock-in-amplifier-based measurement below 7 T [19], and material-characterization systems for magnetic flux densities above 2 T [20]. In addition, Liu et al. [18] proposed a hybrid optomechanical microsystem that couples magnetically sensitive materials, micromechanical resonators, and optical readout, achieving a magnetic-field resolution of 0.1 nT. Overall, these methods provide high-precision reference measurements for magnetic-field calibration. However, they are generally used as offline laboratory calibration platforms rather than as real-time feedback components. Therefore, their direct integration into compact in vivo dose-feedback systems for micro-coil-based neuromodulation remains challenging.

Compared with precision metrology, resonance-enhanced detection is more compatible with magnetic-coupling systems based on micro-coils. By introducing frequency-selective resonant structures, this approach enhances the voltage response to weak magnetic-field signals near the target operating frequency. From a circuit-model perspective, its core mechanism involves a tunable capacitance network that compensates for the reactive component of the probe impedance and minimizes the reactive impedance around resonance.

This exploits the voltage-amplification effect of a high-quality-factor (*Q*-factor) resonant network, thereby improving the weak-field signal-to-noise ratio. Tian et al. [6] developed a magnetic-coupling-resonance-based micro-coil detection method, achieving weak-field measurements from 0 to 3.06 mT with 97.26% accuracy over the 1–120 kHz frequency range. Magnetic-coupling topologies have also been widely investigated in implantable wireless power-transfer systems, including 742 kHz systems with 85% efficiency [63], 3.37 MHz three-coil systems for ocular implants with 62.5% efficiency [64], and 39.86 MHz planar implant coils with 47.2% efficiency [65]. These studies are relevant because the same magnetic-coupling principles that determine wireless power-transfer efficiency also influence magnetic-field distribution, coupling characteristics, and field stability in micro-coil systems. These studies suggest that magnetic-coupling resonance can support power-transfer optimization and provide a theoretical and modeling basis for local magnetic-field estimation and dose evaluation in micro-coil systems.

However, resonance-enhanced detection has inherent limitations. Although a high-*Q* network improves weak-field sensitivity, its narrowband nature makes the measurement response highly sensitive to dynamic detuning under varying coupling, loading, and dielectric conditions. For example, Tian et al. [47] coated the WP-*μ*MS system with an 800 nm Parylene layer to improve biocompatibility. Such encapsulation may alter the effective dielectric environment of the system. Variations in the surrounding biological tissue and microscale spatial misalignment can further change the reflected impedance of the sensing loop. These impedance variations may shift the resonant operating point and distort the amplitude and phase responses of the high-*Q* network, thereby reducing measurement stability. In this context, radio-frequency (RF) near-field resonant probes provide useful references for improving frequency selectivity. Chuang et al. [54] enhanced the magnetic-field response by approximately 8.63 dB at 1.575 GHz by integrating an LC resonant network with a differential-loop probe and an RF balun. Shinde et al. [55] developed a varactor-tuned resonant probe for near-field scanning across 0.9–2.260 GHz. These studies suggest that resonant enhancement can improve sensitivity within the target frequency band. However, maintaining a stable measurement response in multi-frequency and dynamically coupled environments requires adaptive compensation mechanisms, including active reactance compensation, automatic tuning, and online calibration.

### 3.3. Spatial Field Mapping and Hardware-Based Adaptive Tuning

As illustrated in Figure 2c,d, spatial field mapping and hardware-based adaptive tuning represent two complementary system-level approaches. Spatial field mapping characterizes the two- or three-dimensional distribution of localized magnetic fields, whereas adaptive tuning compensates for frequency drift, impedance mismatch, and coupling variations. Their combination can improve both spatial characterization and measurement stability in micro-coil systems.

Spatial field mapping is essential for characterizing microscale magnetic-field distributions and evaluating inter-channel coupling and dose consistency in micro-coil arrays. Three-dimensional scanning can quantify axial attenuation, lateral distribution, and local magnetic-field maxima, while array-based mapping enables the analysis of field overlap and off-target excitation. In RF near-field measurement, Li et al. [53] compared six magnetic-field probes, including single- and double-loop probes covering 0.1–20 GHz and 0.1–5 GHz, respectively. This work provides a useful reference for miniaturized probe design and frequency adaptation in high-frequency magnetic-field measurements. However, most existing spatial mapping techniques are designed for RF or macroscale systems. Extending them to MHz-range near-field mapping of micro-coils remains challenging because of limitations in probe miniaturization and the stringent requirements for spatial resolution.

Array-based TMS magnetic-field measurement provides a useful reference for multichannel synchronous acquisition. Liu et al. [43] developed an FPGA-based 320-channel real-time detection system capable of synchronized measurements within an 8cm×12cm×6cm volume. The system operated over a frequency range of 0–20 Hz and achieved a relative spatial deviation below 2.2%. However, the centimeter-scale dimensions and low operating frequencies of TMS coils limit the direct application of array-based measurement methods to micro-coil magnetic-field detection. Micro-coil systems generate highly localized magnetic fields with stronger spatial gradients and kHz–MHz operating frequencies. Their measurement therefore requires miniaturized probes, precise positioning, and high-frequency synchronous acquisition.

As micro-coil systems evolve toward multi-target and multi-frequency operation, spatial field measurements must account for dynamic coupling and frequency detuning. Reflected impedance, mutual coupling, and load variations are common challenges in resonant magnetic-field measurement and WPT systems. However, in micro-coil arrays, these effects become more consequential because of highly confined magnetic fields and steep spatial gradients. Consequently, fixed-parameter detection or compensation networks may not provide sufficient stability for reliable dose calibration.

Adaptive tuning and active reactance compensation in WPT systems offer useful references. For example, ATAC-based quadrature-phase control has been used to suppress inter-channel coupling and frequency-tolerance effects in a 159.67 kHz multi-receiver system [66]. It has also been extended to 6.78 MHz systems to enable autonomous frequency tuning and improve robustness against frequency drift and multimode resonance [49]. Other hardware-compensation approaches may also provide technical support for dynamic detuning correction in micro-coil systems. Representative examples include disturbance-observer-based phase control in a 400 kHz repeater system [50], full-bridge synchronous series compensation for residual secondary-side reactance at 6.78 MHz [51], and pulse-width modulation (PWM)-controlled switched-capacitor tuning with an inductor-capacitor-capacitor (LCC) inverter at 200 kHz [52]. These studies indicate that active reactance regulation, frequency tracking, and impedance compensation can enhance measurement stability in dynamically coupled, multi-frequency micro-coil systems.

Spatial field measurement for multi-target micro-coil neuromodulation should evolve beyond static three-dimensional scanning. A more integrated framework combining spatial mapping, spectral analysis, multichannel synchronous acquisition, and hardware-based adaptive tuning is therefore desirable. Future closed-loop measurement systems should integrate miniaturized inductive probes, automated scanning, spectral-component extraction, dynamic impedance compensation, and online dose estimation. Such integration could support real-time assessment of magnetic-field distribution, inter-channel coupling, and stimulation dose. Table 1 compares representative electromagnetic measurement methods for high-frequency weak-field detection in micro-coil systems. The comparison considers the upper frequency limit, sensitivity or field response, spatial resolution or probe scale, measurement error or accuracy, main contributions, limitations, applicability, and representative references.

### 3.4. Integrated Measurement and Dose-Calibration Architecture

**Figure 3:**
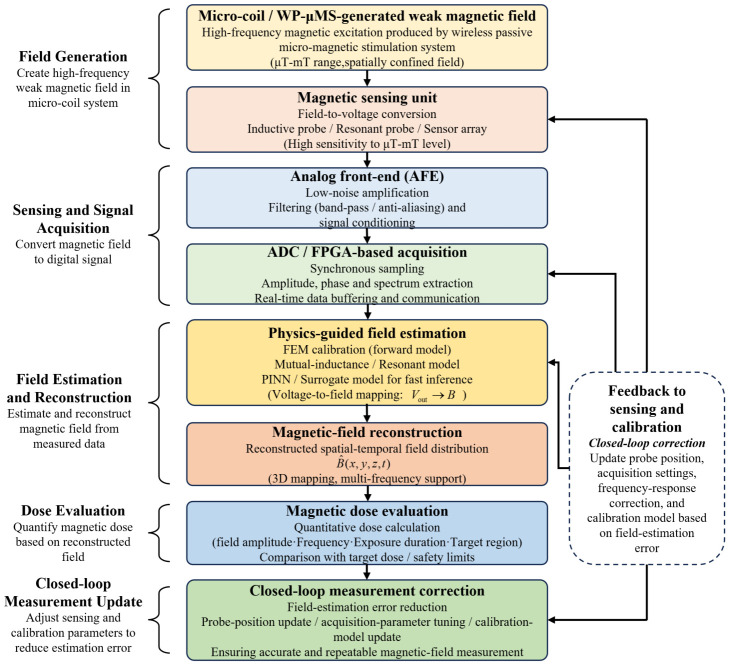
System architecture for high-frequency weak magnetic-field measurement, physics-guided field reconstruction, magnetic-dose evaluation, and closed-loop measurement correction in micro-coil systems.Abbreviations: ADC, analog-to-digital converter; AFE, analog front-end; FEM, finite element method; FPGA, field-programmable gate array; PINN, physics-informed neural network; WP-μMS, wireless passive micro-magnetic stimulation.

Based on the methodological categories discussed in Section 3.1, Section 3.2 and Section 3.3, these techniques can be integrated into a unified measurement-oriented architecture, as illustrated in Figure 3. The architecture includes weak magnetic-field generation, magnetic sensing, an analog front-end (AFE) for signal conditioning, and digital acquisition. It also incorporates physics-guided field estimation, magnetic-field reconstruction, magnetic-dose evaluation, and closed-loop measurement correction. The measured electrical responses are converted into quantitative magnetic-field information through signal acquisition, calibration, and physics-constrained estimation. The feedback path acts on the measurement and calibration processes rather than directly regulating the magnetic-field output of the WP-μMS system. Based on the field-estimation error, the system adjusts the probe position and acquisition parameters and iteratively updates the frequency-response compensation parameters and calibration model. This architecture provides a structured framework for improving measurement accuracy, repeatability, and dose-assessment consistency in high-frequency weak magnetic-field measurements of micro-coil systems.

## 4. Current Challenges and Future Perspectives

Although high-frequency weak magnetic-field detection has advanced in several directions, online calibration of micro-coils for neuromodulation applications remains challenging. In these applications, the required magnetic fields are highly localized, frequency-dependent, dynamically coupled, and often generated by multichannel micro-coil systems. Therefore, future studies should move beyond isolated improvements in sensitivity or spatial resolution. Integrated online calibration frameworks should instead be developed to combine broadband adaptive measurement, component decoupling, and physics-constrained data-driven field estimation.

### 4.1. Broadband Adaptive Measurement and Online Calibration

A major challenge in high-frequency micro-coil measurement is the trade-off between resonant sensitivity and operational stability. High-*Q* resonant probes can enhance weak-field responses. However, their narrowband characteristics make the measurement response vulnerable to dynamic detuning caused by variations in coupling, loading, and dielectric conditions. Broadband adaptive measurement should therefore move beyond simple bandwidth extension and maintain a stable field-to-voltage relationship under changing operating conditions [55].

**Figure 4:**
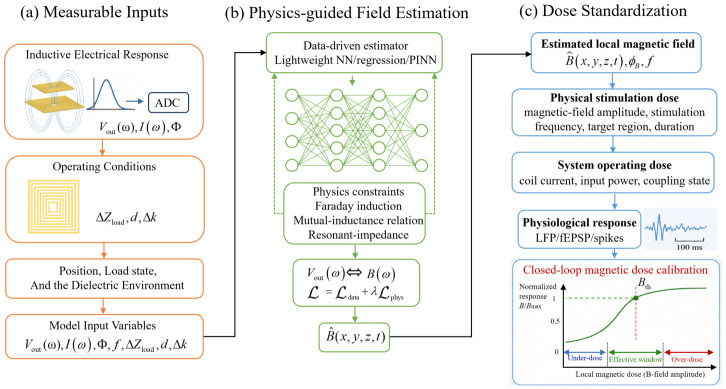
Physics-constrained data-driven framework for voltage-to-field mapping and dose standardization. The framework estimates local magnetic-field strength using electrical parameters and coupling states and establishes dose-standardization relationships among physical dose, system-level dose, and physiological response. It provides a basis for fEPSP/LFP response analysis and closed-loop magnetic-dose calibration. *Note:* The magnetic-field response B(ω) denotes the frequency-domain magnetic field at a given measurement position, and $\phi_{B}$ represents the phase information of the estimated magnetic-field response.

Adaptive tuning and active reactance compensation developed for WPT provide a useful foundation for this direction. Future micro-coil detection systems should move beyond fixed capacitance and offline frequency matching. Instead, they should incorporate real-time impedance tracking, phase correction, and reactance compensation to suppress dynamic detuning. As illustrated in Figure 4a, the measurable inputs for online calibration include the probe output voltage $V_{\mathrm{out}}\left(\omega \right)$, excitation current I(ω), magnetic flux Φ, and operating frequency *f*. Operating-state variables, including load-impedance variation $\Delta Z_{L}$, coupling distance *d*, coupling variation Δk, probe position, load state, and dielectric environment, should also be incorporated into the estimation model. In this framework, the estimation model uses measurable electrical responses and operating-state variables to map system-level responses to local magnetic-field strength and stimulation dose [49,50,66,67,68,69,70].

For reliable dose calibration, researchers should also validate online measurements against high-precision reference methods. High-precision magnetic-field metrology, such as NMR-based calibration and lock-in-amplifier-based flux modulation, may serve as laboratory reference standards for validating online models. Overall, broadband adaptive measurement and online calibration are expected to enhance measurement stability, repeatability, and dose consistency in high-frequency weak-field detection for micro-coil systems [2,7,17,19].

### 4.2. Partial Field Reconstruction and Multichannel Component Decoupling

As micro-coil systems evolve toward multi-target stimulation and array-based architectures, single-point measurements are insufficient to characterize complex magnetic-field distributions [44,45,46,71]. Field overlap between adjacent coils, off-target excitation, and inter-channel mutual coupling require spatial field reconstruction and the decoupling of multichannel components. The physics-guided estimation framework shown in Figure 4b provides the local or spatiotemporal magnetic-field estimate $\hat{B}\left(x,y,z,t\right)$, which can serve as the basis for spatial field reconstruction. For multichannel micro-coil systems, the estimated field should be further decomposed into channel-specific amplitude, phase, and frequency components to distinguish the contribution of each excitation channel to the resultant magnetic-field distribution.

Several studies have demonstrated the feasibility of model-assisted magnetic-field reconstruction. Tian et al. [6] developed a local measurement device based on a mutual-inductance model for millimeter-scale planar square spiral coils, achieving a measurement error of 0.05 mT and an accuracy of 97.26% across 1–120 kHz with a 3.612mm×3.612mm micro-coil structure. This study demonstrates how electromagnetic modeling can improve local magnetic-field estimation beyond direct pointwise measurements and provides a foundation for spatial field reconstruction.

Array-based TMS magnetic-field measurement also provides a useful reference for spatial field reconstruction. Liu et al. [43] demonstrated a 320-channel synchronous magnetic-field measurement system for TMS, showing the feasibility of array-based spatial field reconstruction. However, the macroscopic scale and low operating frequency of these array-based methods prevent their direct application to micro-coil measurements. Such measurements require miniaturized probes, precise positioning, and high-frequency synchronous acquisition. Therefore, future spatial field imaging should integrate miniaturized inductive probes, automated three-dimensional scanning, multichannel lock-in detection, and finite-element model correction. Such an integrated framework would enable microscale magnetic-field reconstruction and quantitative evaluation of dose distribution.

In multi-frequency and multichannel systems, component decoupling is essential for quantitative multi-target stimulation. When different channels overlap spatially or spectrally, the measurement system should distinguish channel-specific amplitude, phase, and intermodulation components rather than measure only the resultant field strength. Researchers can combine spectral filtering, narrowband lock-in detection, and multi-input, multi-output modeling to map each channel’s field contribution to the stimulation dose in the target region. This approach provides a physical basis for closed-loop multichannel neuromodulation.

### 4.3. Model-Data Fusion for Field Estimation and Dose Standardization

Under high-frequency and microscale conditions, reliable dose quantification requires not only accurate magnetic-field measurement but also the establishment of quantitative relationships between electromagnetic exposure and physiological responses. Analytical models can describe mutual inductance, impedance, and resonance behavior, but fabrication tolerances, parasitic effects, spatial misalignment, and heterogeneous tissue environments limit their accuracy. Finite-element simulation can provide detailed spatial field distributions, but its computational cost limits its direct use in real-time closed-loop control. Therefore, model–data fusion approaches provide a potential solution by integrating physical modeling and experimental measurements for quantitative field estimation and dose standardization.

Building upon the adaptive measurement and physics-guided field-estimation frameworks discussed in Section 4.1 and Section 4.2, model–data fusion provides a practical approach for estimating local magnetic fields under complex operating conditions. Finite-element simulations or high-precision calibration measurements can serve as supervised reference data, while measurable electrical responses are combined with physical constraints to improve estimation accuracy and robustness. Lightweight neural networks, regression models, and physics-informed neural networks (PINNs) are potential candidates for implementing such estimation models. The resulting magnetic-field estimate provides the basis for subsequent dose evaluation and standardization. The detailed implementation of physics-guided field estimation, including model inputs, physical constraints, computational complexity, and feasibility for online dose calibration, is further discussed in Section 4.4.

As illustrated in Figure 4c, the estimated local magnetic field serves as the basis for establishing a dose-standardization framework that links physical stimulation parameters, system operating conditions, and physiological responses. The physical stimulation dose is characterized by magnetic-field amplitude, stimulation frequency, target region, and exposure duration. These physical parameters are further associated with system operating conditions, including coil current, input power, and coupling state. Ultimately, the standardized stimulation dose can be linked to physiological responses, such as local field potentials (LFPs), field excitatory postsynaptic potentials (fEPSPs), and neuronal firing activity [72,73]. Experimental studies have demonstrated the importance of establishing quantitative relationships between stimulation dose and physiological responses. Zheng et al. [41] showed that neuronal excitability and long-term potentiation are sensitive to mT-level weak magnetic stimulation, whereas WP-μMS systems have been shown to modulate local field potentials and neuronal firing in hippocampal slices [47]. Together, these studies highlight the importance of establishing quantitative relationships among physical stimulation dose, system operating parameters, and physiological responses to support dose standardization in micro-coil neuromodulation.

The resulting dose–response relationship can further support closed-loop magnetic-dose calibration by updating model parameters, acquisition settings, or calibration coefficients according to the measured physiological responses. Such a framework provides a standardized pathway from high-frequency weak magnetic-field measurement to magnetic-field reconstruction, quantitative dose evaluation, and physiological-response mapping and supports future closed-loop neuromodulation systems.

### 4.4. PINN-Based Field Estimation and Online Dose Calibration

For PINN-based magnetic-field estimation, the model inputs can include measurable electrical responses and operating-state variables, such as probe output voltage, excitation current, operating frequency, magnetic flux response, coupling distance, load variation, and impedance state. The model output can be defined as the estimated local magnetic-field response, including field amplitude or spatial distribution, depending on the measurement objective. The training data can be obtained from finite-element-method (FEM)-based electromagnetic simulations, high-precision calibration experiments, and measured voltage–current responses with different frequencies, coil positions, coupling states, and load conditions.

In this framework, the data loss is used to fit measured or simulated magnetic-field values, whereas the physics loss constrains the model using electromagnetic relationships. PINNs provide a general framework for embedding governing physical laws into neural-network training through physics-residual loss terms [74]. For micro-coil magnetic-field estimation, these constraints may include Faraday induction, mutual-inductance consistency, resonant-impedance relationships, and Maxwell-equation-related electromagnetic constraints. Similar physics-constrained formulations have been applied to magnetostatic, micromagnetic, and generalized electromagnetic-field problems, demonstrating the feasibility of incorporating electromagnetic prior knowledge into neural-field estimation [75,76]. Therefore, PINN-based estimation provides an interpretable alternative to purely data-driven mappings by incorporating electromagnetic constraints into the field-estimation process.

Although PINNs offer a promising approach to physics-constrained magnetic-field estimation, practical implementations must carefully account for their computational overhead. Compared with conventional regression models, PINNs typically incur higher offline training costs because the training process must simultaneously optimize the data-fitting and physics-residual terms. This cost may further increase when dense spatial or frequency-domain collocation points are used. Recent studies on frequency-domain electromagnetic-field simulation and PINN-based surrogate modeling suggest that physics-informed models can be trained offline and then used for fast field inference or real-time simulation [77,78]. Therefore, real-time closed-loop calibration does not require repeatedly solving the full electromagnetic-field problem. After offline training using finite-element simulations, calibration data, and measured electrical responses, a lightweight PINN or physics-constrained neural surrogate could potentially be deployed as a reduced-order estimator for fast online inference. The feasibility of real-time closed-loop dose calibration mainly depends on model size, input dimensionality, inference latency, model compression, and hardware implementation. Lightweight PINNs, compressed neural surrogates, or hybrid FEM–PINN models are more suitable than large-scale full-field PINN solvers for real-time online dose estimation. Therefore, PINN-based approaches are more suitable as fast surrogate estimators after offline training than as real-time solvers of full electromagnetic-field equations.

## 5. Conclusions

High-frequency, weak-magnetic-field detection and dose calibration in micro-coils are essential for implantable micro-magnetic stimulation and closed-loop neuromodulation. This review summarizes the requirements, measurement challenges, methodological advances, and future perspectives of high-frequency weak-field detection and dose calibration in micro-coils. The analysis shows that frequency-dependent probe response, probe-area-induced spatial averaging, weak-field sensitivity limits, multichannel coupling, and dynamic detuning constrain micro-coil magnetic-field measurement. Existing methods, including direct magnetic-field measurement, precision calibration, resonance-enhanced detection, spatial field mapping, and adaptive tuning, each offer distinct advantages but also have inherent limitations. Therefore, existing architectures remain challenging to simultaneously satisfy the requirements for high-frequency response, microscale spatial resolution, online calibration, and multichannel scalability.

To address these limitations, future methodologies should evolve from conventional hardware-level instrumentation toward integrated, intelligence-driven feedback frameworks. Future systems should focus on integrated measurement strategies that combine broadband adaptive measurement, spatial field reconstruction, component decoupling, and PINN-based field estimation. In closed-loop neuromodulation applications, the local magnetic field should be considered not only as a physical output of the device but also as a dose-related parameter. Its quantitative relationship with neural response, stimulation safety, and experimental reproducibility should be carefully established. Establishing a standardized pathway that links high-frequency magnetic-field measurement, online dose calibration, physiological response mapping, and closed-loop control will be important.

By incorporating electromagnetic constraints into learning-based models and integrating them with electrophysiological feedback, such as LFPs and fEPSPs, future systems may achieve more accurate magnetic-field estimation and adaptive dose regulation. Such advances are expected to support the quantitative design and translation of next-generation wireless micro-magnetic stimulation and implantable neuromodulation systems.

## Figures and Tables

**Table 1:** Comparison of representative electromagnetic measurement methods for high-frequency weak-field detection in micro-coil systems.

Method	Frequency/ Upper Limit	Sensitivity/Field Response	Spatial Resolution/ Probe Scale	Typical Error/Accuracy	Main Contribution	Limitation	Applicability	Refs.
Non-resonant inductive probe	0.5–1.5 MHz; up to 13.56 MHz	10–20 mT	Macro-/mm-scale probe	2.5–11.45%	Simple and passive	Parasitics, phase lag, and spatial averaging	Basic detection	[4,5,7,9]
Mutual-inductance correction	1–120 kHz	0–3.06 mT	mm-scale planar coils	0.05 mT; 97.26%	Position-error correction	Model-dependent	Field estimation	[1,6,10,11]
Hall/AMR/TMR sensors	DC–500 kHz	52.17 nT; 0.1477 μT	mm-scale sensors	<0.1%; calibration-dependent	High sensitivity	Limited MHz response	Auxiliary calibration	[8,12,13,14,62]
GMI sensors	Biomagnetic signals: 10–200 Hz; high-frequency excitation	7–$12\;\mathrm{pT}/\sqrt{\mathrm{Hz}}$ noise level	mm-scale amorphous-wire or thin-film probes	Noise-dependent; calibration- and bias-dependent	Room-temperature pT-level weak-field sensing	Sensitive to bias, excitation, and readout conditions	Biomagnetic and magnetic-nanoparticle detection	[57,58,59,60,61]
Precision magnetic metrology	9.038 kHz; pulsed/quasi-static	0.1 nT; μT–T range	Laboratory-scale	Reference-level accuracy	High accuracy	Bulky and costly	Offline reference	[15,16,17,18,19,20]
Resonant magnetic probes	0.1–20 GHz	+8.63 dB response	Near-field loop probes	Not reported	Frequency selectivity	Narrow bandwidth	Design reference	[53,54,55]
MCR-WPT-based estimation	920 kHz–2 MHz; up to 39.86 MHz	0–3.06 mT	mm-/sub-mm-scale coils	0.05 mT; 97.26%	System-compatible	Detuning-sensitive	Highly relevant	[1,2,6,63,64,65]
Array/3D scanning	0–20 Hz; RF up to 20 GHz	System-dependent	$8\times 12\times 6\;{\mathrm{cm}}^{3}$; RF probes	<2.2% spatial deviation	Field mapping	Probe-size limitation	Field imaging	[43,48,53]
Adaptive tuning/ATAC	85 kHz–6.78 MHz	Indirect stabilization	Not a spatial measurement	Indirect stability index	Detuning compensation	Control complexity	Future closed-loop detection	[49,50,51,52,66,67,68,69,70]

Note: Quantitative values are summarized from representative studies when available. Some studies report magnetic-field range, spatial resolution, response enhancement, or spatial deviation rather than noise spectral density in nT/$\sqrt{\mathrm{Hz}}$. For resonant probes, MCR-WPT-based estimation, and adaptive tuning approaches, direct magnetic-field measurement errors are often unavailable. Therefore, reported electrical-response characteristics, frequency-tracking performance, calibration stability, or related system-level metrics are included as indirect indicators of measurement capability.

## Data Availability

The original contributions presented in this study are included in the article. Further inquiries can be directed to the corresponding author.

## Abbreviations

The following abbreviations are used in this manuscript:

ADC	Analog-to-digital converter
AFE	Analog front-end
AMR	Anisotropic magnetoresistive
ATAC	Automatic tuning assist circuit
FEM	Finite element method
fEPSP	Field excitatory postsynaptic potential
FPGA	Field-programmable gate array
LC	Inductor–capacitor
LCC	Inductor–capacitor–capacitor
LFP	Local field potential
LNA	Low-noise amplifier
MCR-WPT	Magnetic-coupling-resonance wireless power transfer
NMR	Nuclear magnetic resonance
PINN	Physics-informed neural network
PWM	Pulse-width modulation
RF	Radio frequency
SQUID	Superconducting quantum interference device
TMR	Tunneling magnetoresistive
TMS	Transcranial magnetic stimulation
WPT	Wireless power transfer
WP-μMS	Wireless passive micro-magnetic stimulation
μMS	Micro-magnetic stimulation
GMI	Giant magnetoimpedance
